# An integrated multimodal model for early prediction of high-risk recurrence phenotype indicative of poor disease-free survival in stage IA NSCLC: a multicenter study

**DOI:** 10.3389/fimmu.2026.1915946

**Published:** 2026-08-06

**Authors:** Linqiang Lai, Yanji Wang, Jiangle Jiang, Dengfa Yang, Jinying Wu, Liangjun Xie, Jiahao Wu, Ruolan Mao, Dengke Zhang, Xiangxue Wang, Wenlong Ming, Jianfei Tu

**Affiliations:** 1 Lishui Central Hospital, The Fifth Affiliated Hospital of Wenzhou Medical University, Lishui, China; 2 Jiangsu Key Laboratory of Intelligent Medical Image Computing, School of Artificial Intelligence, Nanjing University of Information Science and Technology, Nanjing, China; 3 Zhejiang Key Laboratory of Imaging and Interventional Medicine, Lishui Hospital, School of Medicine, Zhejiang University, Lishui, China; 4 Department of Radiology, Taizhou Municipal Hospital, Taizhou, China; 5 Lishui City People’s Hospital, The Sixth Affiliated Hospital of Wenzhou Medical University, Lishui, China; 6 Anhui Key Laboratory of Digital Medicine and Intelligent Health, Bengbu Medical University, Bengbu, China

**Keywords:** adjuvant therapy, computational pathology, disease-free survival, high-risk recurrence phenotype, multimodal model, pathomics, radiomics, stage IA non-small cell lung cancer

## Abstract

**Objectives:**

To develop and validate a preoperative multimodal model that predicts a high-risk recurrence phenotype indicative of poor disease-free survival (DFS), in order to stratify patients with stage IA non-small cell lung cancer (NSCLC).

**Materials and methods:**

This retrospective multicenter study enrolled 342 stage IA NSCLC patients from three independent centers. The high-risk recurrence phenotype (indicative of poor DFS) was defined by postoperative pathology as the presence of spread through air spaces (STAS), lymphovascular invasion (LVI), a predominant solid/micropapillary/complex glandular pattern, or a > 5% solid/micropapillary component. Deep learning features were extracted from preoperative biopsy whole-slide images (WSI) using the UNI foundation model, and CT morphological and textural features were extracted from preoperative chest CT. An early-fusion multimodal model integrating clinical, radiomics, and pathomics features was developed and evaluated with five-fold cross-validation. The Kaplan–Meier method with log-rank tests was used to assess associations between the model-predicted risk and DFS. Logistic regression identified clinical predictors of high-risk pathology. Model interpretability and clinical utility were examined with SHapley Additive exPlanations (SHAP) and calibration analysis, respectively.

**Results:**

The multimodal model achieved higher discriminative performance than each single-modality model in both the internal and external test sets. In the internal test set, it yielded an AUC of 0.76 (95% CI 0.58-0.90); in external validation, AUCs were 0.69 (95% CI 0.55-0.82) in Center 2 and 0.86 (95% CI 0.74-0.96) in Center 3. Multivariable analysis identified solid tumor density as the only independent predictor (OR = 5.13, 95% CI 1.53-17.24; *P* = 0.008). Model-stratified high-risk patients showed significantly inferior DFS in both the training (2-year DFS 78% vs. 99%; log-rank *P* < 0.0001) and external (3-year DFS 86% vs. 100%; log-rank *P* = 0.003) cohorts.

**Conclusion:**

The multimodal model consistently predicted the high-risk recurrence phenotype across multiple centers. This phenotype may serve as a pragmatic indicator of poor DFS to guide earlier individualized treatment decisions, including adjuvant therapy and intensified surveillance, in stage IA NSCLC.

## Introduction

Despite a favorable 5-year overall survival rate of 77-90% following curative resection, stage IA non-small cell lung cancer (NSCLC) harbors substantial heterogeneity in recurrence risk that is not adequately captured by the current TNM-based management (1, 2). The TNM staging system fails to stratify patients with stage IA NSCLC into risk subgroups and lacks the precision required to identify those harboring a high-risk recurrence phenotype; therefore, it does not facilitate tailored treatment decisions. This prognostic ambiguity forces adjuvant therapy into a uniform strategy, risking overtreatment in low-risk individuals and, more importantly, representing a missed therapeutic opportunity for those with a high-risk phenotype who might benefit from intensified intervention. Although disease-free survival (DFS) is the gold-standard endpoint for recurrence assessment, direct prediction of DFS in stage IA NSCLC faces two major challenges: the low recurrence rate leads to a high overfitting risk, and prolonged follow-up combined with adjuvant therapy confounds DFS as an endpoint by altering recurrence timing. Consequently, clinical decision-making requires preoperative qualitative risk stratification (high-risk vs. low-risk) rather than precise time-to-event predictions. Thus, there is an urgent need for a preoperative tool that identifies a high-risk recurrence phenotype strongly associated with DFS, serving as an indicator of poor DFS to guide individualized therapies. The identification of high-risk recurrence phenotypes currently relies on postoperative pathological markers, such as high-grade histologic patterns (solid, micropapillary, or complex glandular patterns), lymphovascular invasion (LVI), and spread through air spaces (STAS), all of which are established independent prognostic factors beyond the TNM system (3–12). LVI is a well-established risk factor in stage IA NSCLC, with a 5-year survival rate below 70-85% (13–17). Similarly, STAS is associated with a higher risk of recurrence, with reported 5-year DFS rates of approximately 70-80% in STAS-positive patients after radical resection (18, 19). Moreover, even a minor component (≥ 5%) of solid/micropapillary patterns adversely impacts outcomes, resulting in a significantly worse 5-year recurrence-free survival (RFS) rate (73% vs. 93%) than in patients without these patterns (20). However, these markers are inherently limited: they are only available postoperatively, precluding preoperative planning; each offers only moderate predictive value in isolation; and they fail to integrate the wealth of preoperatively accessible data from clinical profiles and imaging studies. This underscores the need for a novel methodology that enables early, integrated, and preoperative quantitative risk assessment targeting high-risk recurrence phenotypes.

This study proposes a machine learning-based multimodal integrated model to preoperatively predict the high-risk recurrence phenotype indicative of poor DFS. Specifically, a predictive model was developed by integrating preoperative computed tomography (CT) imaging, clinical baseline data, and preoperative biopsy pathological whole-slide images (WSI) from three independent centers. The high-risk recurrence group was defined based on explicit pathological criteria (STAS, LVI, or solid/micropapillary/complex glandular patterns). Clinical, radiomics, pathomics, and multimodal integrated models were developed and compared. The optimal model was validated in independent external cohorts, and its association with DFS was assessed to establish its clinical prognostic value.

## Materials and methods

This retrospective study was approved by the institutional review boards of the three participating hospitals (approval no. 2025(I)-222-01). The requirement for informed consent was waived. This study adhered to the Declaration of Helsinki and the TRIPOD (Transparent Reporting of a Multivariable Prediction Model for Individual Prognosis or Diagnosis) guidelines (21).

### Study participants

This study enrolled patients with confirmed NSCLC who underwent lobectomy or sublobar resection at three centers: Center 1 (January 1, 2023-December 31, 2023), Center 2 (January 1, 2019-December 31, 2021), and Center 3 (January 1, 2022-December 31, 2023). All had a preoperative chest CT (within four weeks before surgery) and an available preoperative biopsy WSI.

Inclusion criteria: (1) histological diagnosis of NSCLC staged as IA according to the 8th edition of the IASLC TNM staging system (1); (2) availability of preoperative CT and biopsy WSI. Exclusion criteria: (1) poor image quality (inadequate resolution, artifacts, ambiguous features); (2) history of any neoadjuvant therapy prior to surgery. **Table 1** summarizes the baseline characteristics.

**Table 1:** Baseline clinical, CT imaging, and pathological characteristics of the study population.

Characteristic	Training set (Center1; n = 157)		P value (Training set)	Internal test set (Center1; n = 68)		P value (Internal test set)	External test set (Center2; n = 69)		P value (External test set, Center 2)	External test set (Center3; n = 48)		P value (External test set, Center 3)
	Low-risk (n = 135)	High-risk (n = 22)		Low-risk (n = 58)	High-risk (n = 10)		Low-risk (n = 49)	High-risk (n = 20)		Low-risk (n = 44)	High-risk (n = 4)	
Clinical features												
Age (y), median (range)	63(55.0-69.0)	65.5(54.5-69.5)	0.706	60(54.0-69.3)	56(49.3-70.5)	0.467	56(47.0-67.5)	63(55.8-69.8)	0.128	63.5(56.5-70.0)	69(62.3-78.0)	0.173
**Sex, n (%)**			0.582			>0.999			0.599			0.609
Male	59(43.7)	11(50.0)		25(43.1)	4(40.0)		23(46.9)	8 (40.0)		21(47.7)	3 (75.0)	
Female	76(56.3)	11(50.0)		33(56.9)	6(60.0)		26(53.1)	12(60.0)		23(52.3)	1 (25.0)	
**Smoking history, n (%)**			0.039			0.089			0.288			>0.999
Non-smoker	107(79.3)	13(59.1)		49(84.5)	6(60.0)		40(81.6)	14(70.0)		36(81.8)	3 (75.0)	
Smoker	28(20.7)	9 (40.9)		9(15.5)	4(40.0)		9(18.4)	6 (30.0)		8(18.2)	1(25.0)	
**Clinical T stage, n (%)**			0.001			<0.001			<0.001			0.155
T1a	65(48.1)	4 (18.2)		33 (56.9)	0 (0.0)		30 (61.2)	3 (15.0)		14 (31.8)	0 (0.0)	
T1b	56(41.5)	10(45.5)		19(32.8)	5(50.0)		17(34.7)	11(55.0)		23(52.3)	2 (50.0)	
T1c	14(10.4)	8 (36.3)		6(10.3)	5(50.0)		2(4.1)	6(30.0)		7(15.9)	2(50.0)	
CT features												
Consolidation maximum diameter (mm), median (range)	13(10.0-18.0)	19.5(12.8-23.3)	0.002	14(9.8-19.0)	18.5(15.0-25.8)	0.017	10.0(7.0-13.5)	16.5(13.0-23.8)	<0.001	13.8(9.1-18.0)	19(14.4-24.0)	0.126
**Density, n (%)**			<0.001			0.022			0.174			<0.001
Non-solid	30(22.2)	1(4.5)		15(25.9)	0(0.0)		18(36.7)	3(15.0)		11(25.0)	0(0.0)	
Part-solid	72(53.3)	4(18.2)		27(46.6)	3(30.0)		23(46.9)	10(50.0)		33(75.0)	3(75.0)	
Solid	33(24.5)	17(77.3)		16(27.5)	7(70.0)		8(16.4)	7(35.0)		0(0.0)	1(25.0)	
**Lobulated sign, n (%)**			0.127			>0.999			>0.999			0.111
Negative	26(19.3)	1(4.5)		16(27.6)	2(20.0)		7(14.3)	2(10.0)		23(52.3)	0(0.0)	
Positive	109(80.7)	21(95.5)		42(72.4)	8(80.0)		42(85.7)	18(90.0)		21(47.7)	4(100.0)	
**Spiculated sign, n (%)**			0.122			0.729			0.391			0.012
Negative	54(40.0)	5(22.7)		24(41.4)	3(30.0)		30(61.2)	10(50.0)		31(70.5)	0(0.0)	
Positive	81(60.0)	17(77.3)		34(58.6)	7(70.0)		19(38.8)	10(50.0)		13(29.5)	4(100.0)	
**Pleural indentation sign, n (%)**			0.033			0.294			0.001			0.069
Negative	59(43.7)	4(18.2)		25(43.1)	2(20.0)		39(79.6)	8(40.0)		33(75.0)	1(25.0)	
Positive	76(56.3)	18(81.8)		33(56.9)	8(80.0)		10(20.4)	12(60.0)		11(25.0)	3(75.0)	
**Vacuolar sign, n (%)**			0.488			>0.999			0.675			>0.999
Negative	41(30.4)	6(27.3)		26(44.8)	4(40.0)		39(79.6)	15(75.0)		35(79.5)	3(75.0)	
Positive	94(69.6)	16(72.7)		32(55.2)	6(60.0)		10(20.4)	5(25.0)		9(20.5)	1(25.0)	
**Vascular convergence sign, n (%)**			0.033			0.475			0.770			0.480
Negative	59(43.7)	4(18.2)		23(39.7)	2(20.8)		36(73.5)	14(70.0)		38(86.4)	3(75.0)	
Positive	76(56.3)	18(81.8)		35(60.3)	8(80.0)		13(26.5)	6(30.0)		6(13.6)	1(25.0)	
Pathological features												
**Differentiation grade, n (%)**			<0.001			0.002			<0.001			0.014
Poor	5(3.7)	5(22.7)		2(3.4)	3(30.0)		1(2.0)	5(25.0)		1(2.3)	2(50.0)	
Middle	41(30.4)	13(59.1)		19(32.8)	5(50.0)		16(32.7)	11(55.0)		16(36.4)	1(25.0)	
High	89(65.9)	4(18.2)		37(63.8)	2(20.0)		32(65.3)	4(20.0)		27(61.3)	1(25.0)	
**High-grade histological subtype, n (%)**			<0.001			0.147			0.199			0.083
Negative	135(100.0)	19(86.4)		58(100.0)	9(90.0)		49(100.0)	18(90.0)		44(100.0)	3(75.0)	
Positive	0(0.0)	3(13.6)		0(0.0)	1(10.0)		0(0.0)	2(10.0)		0(0.0)	1(25.0)	

Bold values indicate statistically significant differences (P < 0.05).

Patients from Center 1 were randomly split 7:3 into training and internal test sets by stratified sampling. Data from Centers 2 and 3 formed an external test cohort.

### CT image acquisition

Center 2 and 3: GE 16-slice CT (BrightSpeed 16, GE Healthcare, USA). Plain scan, 120 kVp, SmartmA (200–300 mA). Supine, craniocaudal, thoracic entrance to lung base. Gantry rotation 0.5 s, collimation 16 × 0.625 mm, pitch 1.375:1. Reconstructions: lung (1.25 mm) and soft tissue (5 mm) with appropriate intervals; lung and standard soft-tissue algorithms. Field of view (FOV) 35–40 cm, matrix 512 × 512.

Center 1: Siemens 16-slice CT (SOMATOM go, Siemens Healthineers). 120 kVp, CareDose 4D (150–200 mAs), same position and range. Rotation 0.6 s, collimation 16 × 0.6 mm, pitch 0.8:1. Reconstructions: lung (1.0 mm) and soft tissue (5 mm) with B70f (lung) and B30f (soft-tissue) kernels. FOV 36–40 cm, matrix 512 × 512.

Lung-window sequences were reconstructed at 1–1.25 mm. Two thoracic radiologists, independently and blinded to each other, assessed: maximum diameter; nodule type (non-solid, part-solid, or solid); margin (smooth, lobulated, or irregular/spiculated); air bronchogram; cavity/vacuole; pleural indentation; vessel convergence. Disagreements were resolved by a third radiologist, all blinded to clinical and pathological results.

### Histological evaluation and definition of the high-risk recurrence phenotype

Pathological stage was determined by the 8th TNM classification (1). The high-risk recurrence phenotype was defined on postoperative specimens as at least one of the following: STAS; LVI; a predominant solid, micropapillary, or complex glandular pattern; or more than 5% solid/micropapillary component. Otherwise, the case was classified as low risk. The STAS definition followed the World Health Organization (WHO) classification (11). LVI was confirmed by hematoxylin and eosin (HE) staining or immunohistochemistry. The predominant pattern followed the IASLC consensus (3). Two thoracic pathologists, blinded to clinical and imaging data, evaluated all specimens; a third resolved disagreements. The multimodal model used preoperative biopsy WSI as input, while the high-risk label was derived from postoperative pathology.

### Model construction and validation

This study built three single-modality models (clinical, radiomics, pathomics) and then a multimodal model via feature-level concatenation, as detailed in the Appendix (**Supplementary Material**).

### Clinical model development

The clinical prediction model was constructed using 16 preoperative variables (demographics and CT morphological features). After cleaning and median imputation of numeric features, Z-score standardization was applied exclusively on the training set to prevent information leakage. An L2-regularized logistic regression identified non-zero coefficient features as the final signature, which were then used to train a radial basis function (RBF) kernel support vector machine (SVM) for baseline risk prediction. Binary encoding was applied: high-risk recurrence = 1; low-risk = 0. Detailed preprocessing steps and parameter settings are provided in the **Supplementary Material**.

### CT radiomics model development

The CT radiomics model was built on quantitative features extracted from preoperative CT images. Tumor regions of interest (ROI) were manually delineated, and features were extracted using Pyradiomics (v3), covering seven categories: first-order statistics, shape features, Gray Level Co-occurrence Matrix (GLCM), Gray Level Run Length Matrix (GLRLM), Gray Level Size Zone Matrix (GLSZM), Gray Level Dependence Matrix (GLDM), and Neighboring Gray Tone Difference Matrix (NGTDM). The dataset was split into training (70%) and test (30%) sets via patient-level stratified random sampling (seed = 42). Z-score normalization was applied exclusively on the training set, and Gaussian noise (mean = 0, SD = 0.1) was added for data augmentation. An RBF-kernel SVM classifier was used for risk prediction. Detailed preprocessing steps and parameter settings are provided in the **Supplementary Material**.

### Pathology (WSI) model development

The pathology model leveraged deep learning features from preoperative biopsy WSIs. Foreground tissue was segmented from HE-stained slides, tessellated into 256 × 256 pixel patches at 20× magnification, and processed through the pre-trained UNI foundation model to extract high-dimensional embeddings. The dataset was split patient-wise into training (70%) and test (30%) sets via stratified random sampling, ensuring no data leakage. Patch-level features underwent Z-score normalization (scaler fitted on training patches only). An RBF-kernel SVM classifier was trained on the standardized features; for patient-level inference, patch probabilities were averaged, and a 0.5 threshold was applied for binary risk prediction. Detailed preprocessing steps and parameter settings are provided in the **Supplementary Material**.

### Multimodal integrated model construction

A multimodal model was constructed via feature-level concatenation (see **Supplementary Material**). For each patient, clinical and radiomics features were tiled to match the number of patches and then concatenated with enhanced WSI features (original patch-level WSI features concatenated with log-transformed absolute value features) to form the final patch-level multimodal fusion features. To reduce the dimensionality of high-dimensional multimodal features while preserving predictive information, a logistic-regression-based proxy selection strategy was applied, with all steps performed exclusively on the training set (and repeated within each cross-validation fold) to avoid information leakage. A logistic regression proxy was trained on a random subset of fused features (max 200,000 samples) to rank patches by predicted risk; the top 500 patches per patient were retained. Sampled features were Z-score normalized (training-fitted), and an SVM with RBF kernel was trained. A logistic regression calibrator converted decision values to calibrated probabilities. Patient-level risk probability was averaged from calibrated patch probabilities, and a pre-specified decision threshold was applied to yield binary classification. Model discrimination was assessed by receiver operating characteristic (ROC) curves, area under the ROC curve (AUC) with 95% confidence interval (CI), confusion matrices, accuracy, sensitivity, specificity, precision, F1-score, area under the precision-recall curve (PR-AUC), and balanced accuracy.

### Model interpretability analysis

To address the “black-box” nature of machine learning algorithms and enhance clinical transparency, we employed the SHapley Additive exPlanations (SHAP) framework. Specifically, a Kernel Explainer was used to quantify the marginal contribution of each feature (clinical, radiomics, and pathomics) to the fusion model’s final prediction. This analysis provides global feature importance rankings, allowing for the visualization of key biomarkers that drive risk stratification decisions, thereby facilitating the clinical understanding of the model’s logic.

### Statistical analysis

Statistical analyses and model development used Python 3.10 and Scikit-learn 1.3.0; the full code pipeline is summarized in the **Supplementary Material**. The development cohort (Center 1) was split 70/30 into training and internal test sets by stratified sampling, preserving class distribution. Hyperparameter tuning and preliminary evaluation were performed only on the training set; the internal test set was used for final validation. No patient overlap existed between training, internal test, and external cohorts (Centers 2 and 3), and no temporal overlap was observed, eliminating data leakage. Z-score standardization and median imputation of missing clinical values were fitted exclusively on the training set (repeated within cross-validation folds) and then applied to test/validation sets. To mitigate overfitting in the radiomics model, data augmentation was applied to the training set by injecting Gaussian noise. Four SVM-RBF models (clinical, radiomics, pathomics, multimodal) were developed, all using class_weight = “balanced” to address class imbalance. Model discrimination was assessed by AUC with 95% CI (bootstrap, 2000 iterations), accuracy, sensitivity, specificity, precision, F1-score, PR-AUC, and balanced accuracy. Confusion matrices (absolute counts and percentages) were provided for all models. Sensitivity analyses with different random seeds and thresholds confirmed stability. The classification threshold was determined by the Youden index on the training set and applied uniformly across all cohorts. Calibration was evaluated using calibration curves, Brier score, and expected calibration error (ECE). SHAP (Kernel Explainer) values were calculated for interpretability. Survival analysis (Kaplan–Meier with log-rank test) assessed DFS in the Center 1 cohort stratified by model-predicted risk. Median follow-up, recurrence events, and censoring rates were reported. All tests were two-sided; *P* < 0.05 was considered significant.

## Results

### Participant characteristics

**Figure 1:**
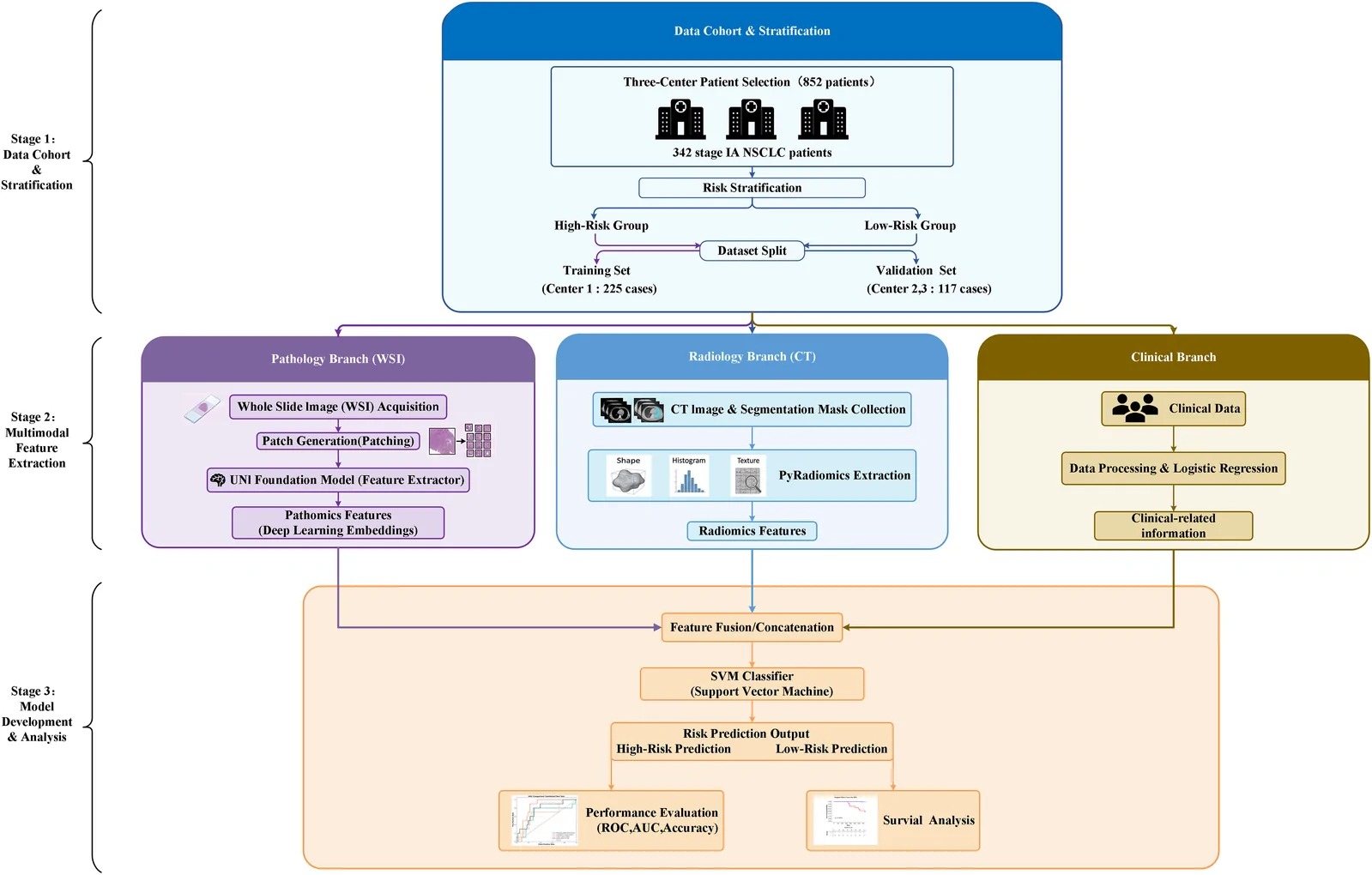
Study design and pipeline. CT, computed tomography; AUC, area under the receiver operating characteristic curve; ROC, receiver operating characteristic; SVM, support vector machine; WSI, whole-slide images.

**Figure 2:**
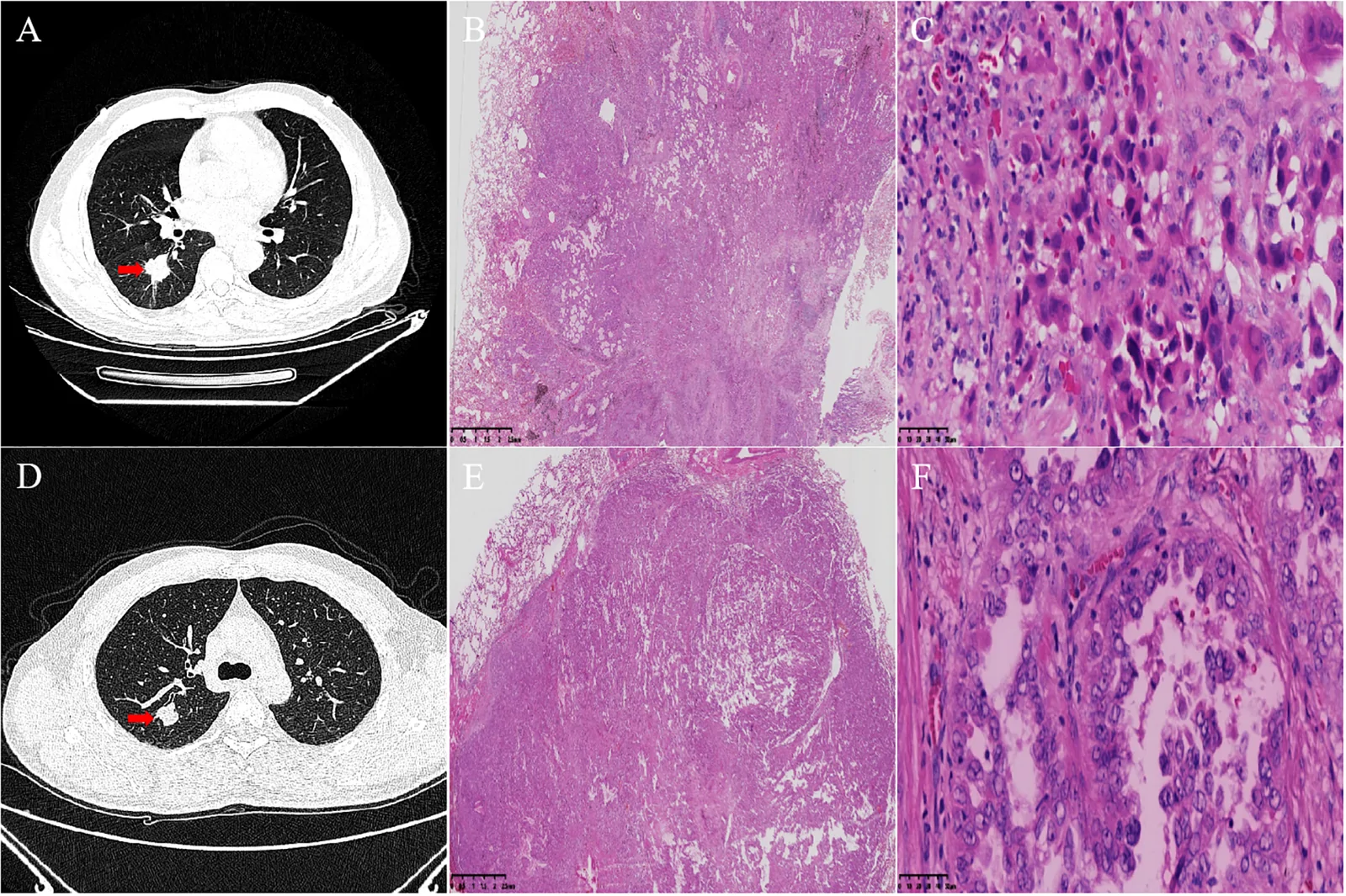
Radiopathologic features (CT and WSI) of stage IA NSCLC cases in the predicted high-risk versus low-risk recurrence phenotype groups. Low-risk phenotype group (59-year-old male): **(A)** CT shows a 3-cm solid nodule in the right lower lobe (red arrow). **(B)** HE (original magnification ×10) shows diffuse infiltration with ill-defined borders. **(C)** HE (higher magnification ×400) reveals marked atypia, hyperchromatic nuclei, and abundant cytoplasm. High-risk phenotype group (43-year-old male): **(D)** CT shows a 2.5-cm solid nodule in the right upper lobe (red arrow). **(E)** HE (original magnification ×10) shows diffuse infiltration with glandular and papillary structures. **(F)** HE (higher magnification ×400) reveals marked atypia, coarse chromatin, and vesicular nuclei. Both cases were diagnosed as lung adenocarcinoma. CT, computed tomography; WSI, whole-slide images; NSCLC, non-small cell lung cancer; HE, hematoxylin-eosin.

A total of 852 patients from three centers were screened; 342 with stage IA NSCLC met the inclusion criteria (**Figure 1**). Based on postoperative pathology, 56 were classified as high-risk and 286 as low-risk for recurrence phenotype (defined by STAS, LVI, predominant solid/micropapillary/complex glandular pattern, or more than 5% solid/micropapillary component) (**Figure 2**). The high-risk phenotype group showed more advanced features: higher T1c stage, larger solid components, pleural indentation, vascular convergence, and poorer differentiation or high-grade subtypes (**Table 1**). Baseline characteristics were well balanced between the training and test sets.

### Univariate and multivariable analyses for high-risk recurrence phenotype prediction

In the training set, six variables demonstrated a univariate association with high-risk recurrence pathological status (*P* < 0.10): smoking history, clinical T stage, consolidation maximum diameter, tumor density, pleural indentation, and vascular convergence. Subsequent multivariable logistic regression analysis revealed that tumor density (solid vs. non- or part-solid) was the only remaining independent predictor, with an adjusted odds ratio (OR) of 5.13 (95% CI 1.53-17.24; *P* = 0.008). The complete regression results are shown in **Table 2**.

**Table 2:** Univariable and multivariable logistic regression analysis of factors associated with the high-risk recurrence phenotype in the training set.

Variable	Univariable analysis		Multivariable analysis	
	OR (95% CI)	P value	OR (95% CI)	P value
Age	1.01 (0.97-1.06)	0.562		
Sex (male vs. female)	1.29 (0.52-3.18)	0.582		
Smoking history (yes vs. no)	2.65 (1.06-6.80)	0.044	2.23 (0.76-6.87)	0.140
Clinical T stage (T1c vs. T1a + T1b)	4.94 (1.76-13.83)	0.002	1.90 (0.54-6.64)	0.315
Consolidation maximum diameter (per mm)	1.51 (0.94-2.44)	0.088	1.07 (0.66-1.75)	0.775
Density (solid vs. non- or part-solid)	5.16 (1.65-15.87)	0.005	5.13 (1.53-17.24)	0.008
Lobulated sign (yes vs. no)	5.01 (0.64-38.96)	0.124		
Spiculated sign (yes vs. no)	2.27 (0.79-6.51)	0.128		
Pleural indentation sign (yes vs. no)	3.49 (1.12-10.87)	0.031	3.01 (0.87-10.41)	0.082
Vacuolar sign (yes vs. no)	1.43 (0.52-3.88)	0.489		
Vascular convergence sign (yes vs. no)	3.49 (1.12-10.87)	0.031	3.40 (0.99-11.66)	0.052

### Development and performance of predictive models

Four predictive models were developed and evaluated: a clinical model, a radiomics model, a pathomics model, and a multimodal integrated model. The discriminative performance of each model across the training, internal and external test sets is summarized in **Table 3**.

**Table 3:** Performance comparison of pathomics (WSI), radiomics (CT), clinical, and multimodal fusion models across the training, internal test, and external test sets.

Model	AUC (95% CI)	Accuracy (95% CI)	Sensitivity (95% CI)	Specificity (95% CI)	F1 score (95% CI)
Internal test set (Center 1)					
Pathomics (WSI) model	0.65(0.41-0.86)	0.82 (0.73-0.91)	0.10(0.00-0.33)	0.95 (0.89-1.00)	0.14(0.00-0.42)
Radiomics (CT) model	0.83(0.73-0.92)	0.78 (0.68-0.88)	0.70(0.38-1.00)	0.79 (0.68-0.89)	0.48(0.24-0.69)
Clinical model	0.67(0.47-0.85)	0.75 (0.65-0.85)	0.70(0.38-1.00)	0.76 (0.64-0.87)	0.45(0.21-0.65)
**Multimodal fusion model**	0.76(0.58-0.90)	0.85 (0.76-0.93)	0.20(0.00-0.50)	0.97 (0.91-1.00)	0.29(0.00-0.60)
External test set (Center 2)					
Pathomics (WSI) model	0.52(0.36-0.66)	0.71 (0.60-0.81)	0.00(0.00-0.00)	1.00 (1.00-1.00)	0.00(0.00-0.00)
Radiomics (CT) model	0.68(0.52-0.82)	0.71 (0.61-0.81)	0.00(0.00-0.00)	1.00 (1.00-1.00)	0.00(0.00-0.00)
Clinical model	0.62(0.47-0.77)	0.71 (0.61-0.83)	0.00(0.00-0.00)	1.00 (1.00-1.00)	0.00(0.00-0.00)
**Multimodal fusion model**	0.69(0.55-0.82)	0.68 (0.57-0.78)	0.65(0.43-0.85)	0.69 (0.55-0.82)	0.54(0.35-0.69)
External test set (Center 3)					
Pathomics (WSI) model	0.85(0.74-0.95)	0.81 (0.69-0.92)	1.00(1.00-1.00)	0.79 (0.66-0.91)	0.47(0.15-0.73)
Radiomics (CT) model	0.79(0.22-1.00)	0.91 (0.83-0.98)	0.00(0.00-0.00)	1.00 (1.00-1.00)	0.00(0.00-0.00)
Clinical model	0.84(0.69-1.00)	0.91 (0.83-0.98)	0.00(0.00-0.00)	1.00 (1.00-1.00)	0.00(0.00-0.00)
**Multimodal fusion model**	0.86(0.74-0.96)	0.81 (0.69-0.92)	1.00(1.00-1.00)	0.80 (0.67-0.90)	0.47(0.15-0.75)

AUC, area under the receiver operating characteristic curve; CI, confidence interval; CT, computed tomography; OR, odds ratio; WSI, whole-slide images.

Bold values indicate the best performance among all compared models for each metric.

**Figure 3:**
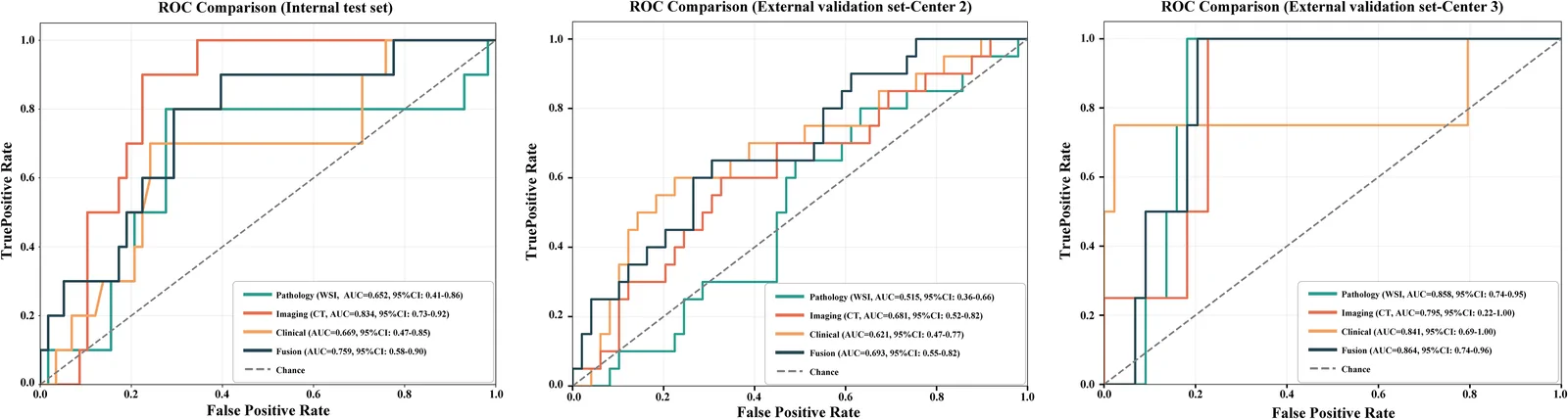
Receiver operating characteristic (ROC) curves of the four predictive models (clinical, radiomics, pathomics, and multimodal) in the training set, internal test set, and two external test sets.

The multimodal integrated model demonstrated the highest discriminative performance in both the internal and external test sets. In the independent internal test set (30% hold-out from Center 1), it achieved an AUC of 0.76 (95% CI 0.58-0.90). Critically, the model exhibited favorable predictive performance across two external cohorts, with AUCs of 0.69 (95% CI 0.55-0.82) in Center 2 and 0.86 (95% CI 0.74-0.96) in Center 3 (**Figure 3**). The multimodal integrated model achieved the best overall performance across key metrics (accuracy, sensitivity, specificity, F1-score) in both internal and external test sets, as detailed in **Table 3**.

### Survival analysis based on risk stratification

**Figure 4:**
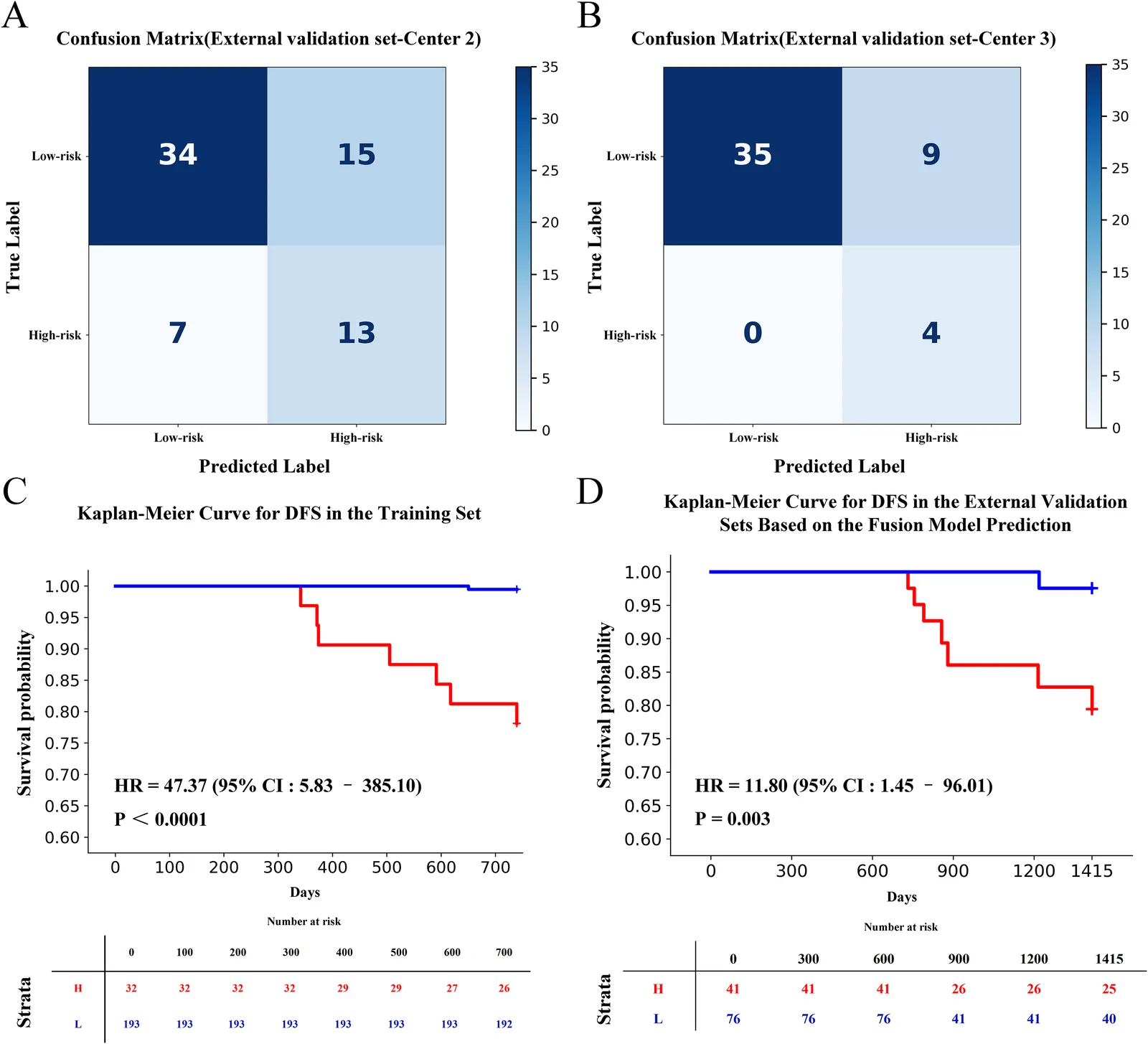
Corresponding confusion matrix and Kaplan-Meier curves for disease-free survival (DFS) in the high- and low-risk recurrence phenotype groups. **(A)** Confusion matrix for Center 2. **(B)** Confusion matrix for Center 3. **(C)** DFS in the training set. **(D)** DFS in the external validation set. DFS, disease-free survival.

In the training set, after a median follow-up of 28 months (interquartile range: 25-31), 6 of 157 patients (3.8%) experienced disease recurrence, and the remaining 151 (96.2%) patients were censored. Based on Kaplan-Meier analysis, the model-predicted high-risk recurrence phenotype group exhibited significantly lower DFS rates than the low-risk recurrence phenotype group in both the training (2-year: 78% vs. 99%; log-rank *P* < 0.0001) and external validation (3-year: 86% vs. 100%; log-rank *P* = 0.003) cohorts. Kaplan-Meier 2-year DFS curves and the confusion matrix are shown in **Figure 4**.

### Model interpretation and calibration

**Figure 5:**
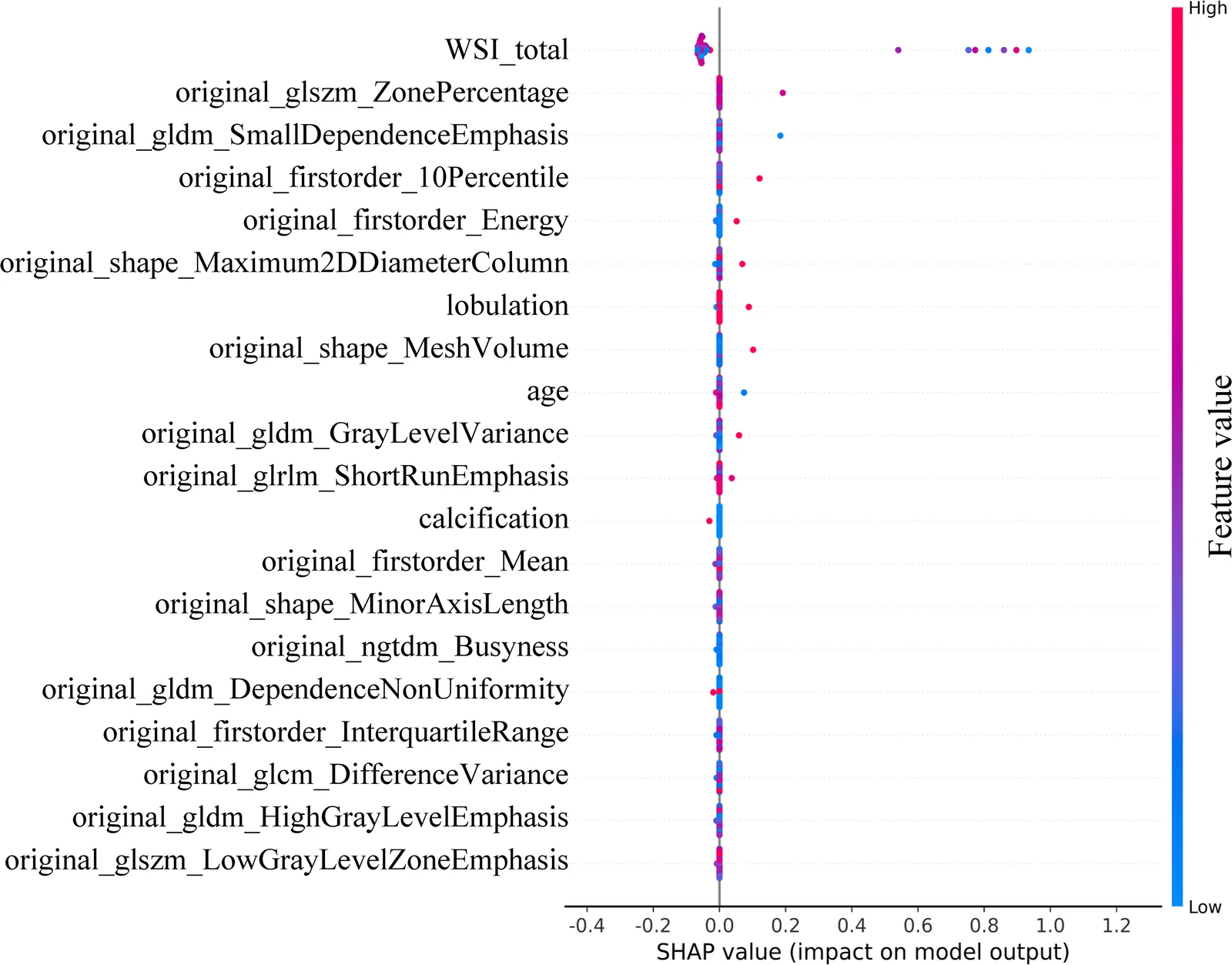
SHAP analysis for interpretation of the multimodal fusion model. The plot shows global feature importance rankings, with the three most influential features being WSI_total, original_glszm_ZonePercentage, and original_gldm_SmallDependenceEmphasis. SHAP, SHapley Additive exPlanations; WSI, whole-slide images.

**Figure 6:**
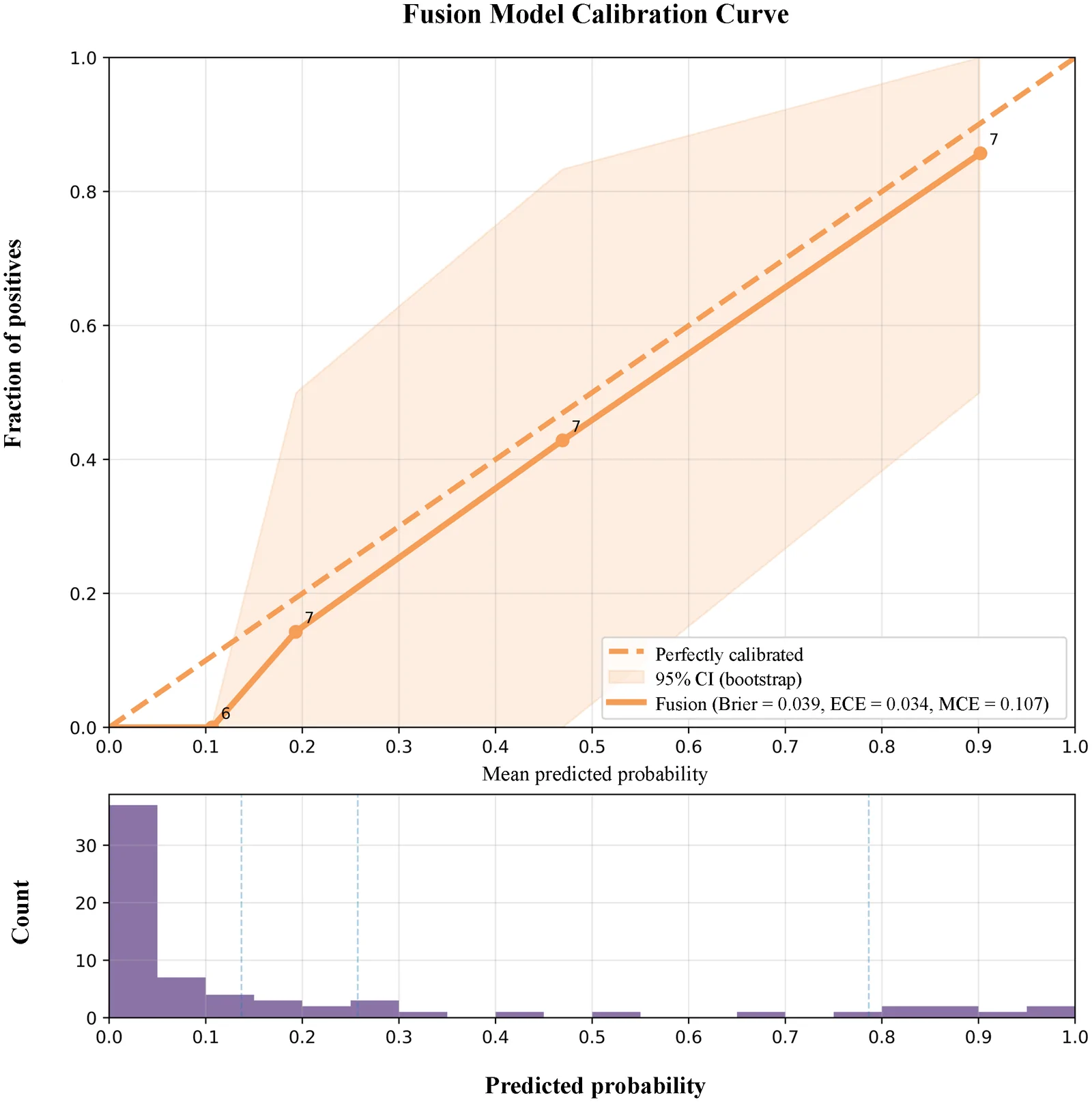
Calibration curves of the multimodal fusion model in the training, internal test, and two external test sets. The curves closely approximate the diagonal, indicating good calibration. Quantitative metrics: Brier score = 0.039, ECE = 0.034, MCE = 0.107.

SHAP analysis identified the three most influential features in the fusion model: WSI_total, original_glszm_ZonePercentage, and original_gldm_SmallDependenceEmphasis (**Figure 5**). Calibration curves closely approximated the diagonal across all datasets (**Figure 6**). Quantitative metrics confirmed good calibration: Brier score = 0.039, ECE = 0.034, MCE = 0.107. The Hosmer-Lemeshow goodness-of-fit test yielded non-significant results (*P* > 0.05), indicating no substantial deviation from perfect calibration.

## Discussion

Despite favorable overall prognosis, stage IA NSCLC exhibits considerable heterogeneity in recurrence risk that is not adequately captured by current TNM-based management (1, 2). Low recurrence rates and adjuvant therapy confounding limit direct DFS prediction in stage IA NSCLC; therefore, a high-risk recurrence phenotype indicative of poor DFS was used to guide surgical extent and adjuvant therapy. To preoperatively predict this phenotype, this study developed and validated a multimodal fusion model integrating CT, biopsy WSI, and clinical data. The model outperformed all single-modality models, achieving an AUC of 0.76 (95% CI 0.58-0.90) in the internal test set and 0.69 (95% CI 0.55-0.82) and 0.86 (95% CI 0.74-0.96) in two independent external cohorts. Model-based risk stratification was prognostic: high-risk patients had significantly lower DFS in both the training (2-year: 78% vs. 99%; log-rank *P* < 0.0001) and external validation (3-year: 86% vs. 100%; log-rank *P* = 0.003) cohorts. Multivariable analysis identified solid tumor density as a only independent predictor (OR = 5.16, 95% CI 1.65-15.87, *P* = 0.008), consistent with prior studies linking solid CT appearance to aggressive pathology and higher recurrence risk (22, 23). By integrating complementary multimodal information, the fusion model enables preoperative identification of high-risk recurrence patients for timely intervention.

The fusion model’s superior performance is explained by the complementary and biologically congruent features captured from each modality. The strong CT-based association between solid density and high-risk pathology (OR = 5.16, 95% CI 1.65-15.87; *P* = 0.008) is consistent with literature linking this imaging phenotype to aggressive histology (20). Applying the pre-trained UNI model to WSI enabled extraction of sub-visual morphometric features (e.g., architectural disorder, cellular spatial patterns, and stromal reactions) that may escape standard review. Deep analysis quantified subtle patterns biologically linked to metastatic competence, including STAS, supporting evidence that computational pathology reveals prognostically relevant information beyond conventional assessment (24, 25). Critically, fusing these deep pathological features with congruent CT phenotypes and clinical context (e.g., smoking history) creates a biologically coherent risk profile. This synergy underpins the model’s enhanced accuracy and clinical relevance, as shown by the significantly inferior DFS in the model-predicted high-risk group (training: 78% vs. 99%; external validation: 86% vs. 100%; both *P* < 0.05) and by decision curve analysis demonstrating net clinical benefit. This model therefore operationalizes a multi-scale biological rationale, integrating *in vivo* imaging with ex vivo microarchitecture to provide a more reliable and actionable stratification tool than single-modality assessments.

This finding has important clinical implications for stage IA NSCLC management. Whether high-risk patients should receive adjuvant therapy remains controversial; current guidelines do not routinely recommend it despite evidence of poorer outcomes in selected subgroups (26–29). The present model resolves this dilemma by accurately identifying patients with a truly high-risk recurrence phenotype. Using routine HE-stained WSI, the UNI model provides a continuous risk score that, when integrated with CT radiomics and clinical data, offers superior stratification compared with unimodal approaches. For high-risk patients, model predictions can guide adjuvant chemotherapy decisions and intensified surveillance; for low-risk patients, observation alone avoids unnecessary toxicity and less frequent follow-up. Thus, this risk-stratified approach enables personalized postoperative management, optimizing therapeutic intervention and resource allocation based on individual recurrence probability.

This study has several limitations. First, the retrospective design may have introduced selection bias. Second, the small high-risk recurrence group (n = 56) could affect model stability and survival analysis power; pooling data from Centers 2 and 3 helped mitigate this. Third, inter-center variations in pathological slide preparation and CT protocols may affect feature reproducibility. Fourth, differences in surgical and follow-up timelines across centers precluded uniform short-term DFS assessment, although longer observation revealed significant divergence. Fifth, the inherent “black-box” nature of deep learning limits full biological interpretability. Sixth, the high-risk recurrence phenotype is a pragmatic indicator of poor DFS in stage IA NSCLC and requires larger prospective validation.

In conclusion, the present model integrates preoperative CT, biopsy WSI, and clinical data to objectively stratify recurrence risk and potentially personalize postoperative management for stage IA NSCLC. By preoperatively identifying patients harboring a high-risk recurrence phenotype, this approach may inform decisions on adjuvant therapy, including emerging immune-based strategies, and support intensified surveillance, thereby bridging the gap between TNM staging and individualized immuno-oncology management.

## Data Availability

The datasets presented in this study can be found in online repositories. The names of the repository/repositories and accession number(s) can be found in the article/**Supplementary Material**.

## References

[1] Goldstraw P Chansky K Crowley J Rami-Porta R Asamura H Eberhardt WE . The IASLC lung cancer staging project: proposals for revision of the TNM stage groupings in the forthcoming (eighth) edition of the TNM classification for lung cancer. J Thorac Oncol. (2016) 11:39–51. doi: 10.1016/j.jtho.2015.09.009 26762738

[2] Maeda R Ishii G Yoshida J . Influence of cigarette smoking on histological subtypes of stage I lung adenocarcinoma. J Thorac Oncol. (2011) 6:743–50. doi: 10.1097/JTO.0b013e3182103714 21325977

[3] Travis WD Brambilla E Noguchi M Nicholson AG Geisinger KR Yatabe Y . International Association for the Study of Lung Cancer/American Thoracic Society/European Respiratory Society international multidisciplinary classification of lung adenocarcinoma. J Thorac Oncol. (2011) 6:244–85. doi: 10.1097/JTO.0b013e318206a221 21252716 PMC4513953

[4] Talvitie EM Vilhonen H Kurki S Karlsson A Orte K Almangush A . High tumor mutation burden predicts favorable outcome among patients with aggressive histological subtypes of lung adenocarcinoma: a population-based single-institution study. Neoplasia. (2020) 22:333–42. doi: 10.1016/j.neo.2020.05.004 32585428 PMC7317687

[5] Kadota K Kushida Y Kagawa S Ishikawa R Ibuki E Inoue K . Cribriform subtype is an independent predictor of recurrence and survival after adjustment for the eighth edition of TNM staging system in patients with resected lung adenocarcinoma. J Thorac Oncol. (2019) 14:245–54. doi: 10.1016/j.jtho.2018.09.028 30336325

[6] Collier FC Blakemore WS Kyle RH Enterline HT Kirby CK Johnson J . Carcinoma of the lung: factors which influence five year survival with special reference to blood vessel invasion. Ann Surg. (1957) 146:417–23. doi: 10.1097/00000658-195709000-00010 13459291 PMC1450462

[7] Neri S Yoshida J Ishii G Matsumura Y Aokage K Hishida T . Prognostic impact of microscopic vessel invasion and visceral pleural invasion in non-small cell lung cancer: a retrospective analysis of 2657 patients. Ann Surg. (2014) 260:383–8. doi: 10.1097/SLA.0000000000000617 24646539

[8] Shoji F Haro A Yoshida T Ito K Morodomi Y Yano T . Prognostic significance of intratumoral blood vessel invasion in pathologic stage IA non-small cell lung cancer. Ann Thorac Surg. (2010) 89:864–9. doi: 10.1016/j.athoracsur.2009.09.047 20172144

[9] Zhang Y Sun Y Xiang J Zhang Y Chen H . A clinicopathologic prediction model for postoperative recurrence in stage IA non-small cell lung cancer. J Thorac Cardiovasc Surg. (2014) 148:1193–9. doi: 10.1016/j.jtcvs.2014.02.064 24667022

[10] Shimada Y Saji H Yoshida K Kakihana M Honda H Nomura M . Pathological vascular invasion and tumor differentiation predict cancer recurrence in stage IA non-small cell lung cancer after complete surgical resection. J Thorac Oncol. (2012) 7:1263–70. doi: 10.1097/JTO.0b013e31825cca6e 22673056

[11] Travis WD Brambilla E Nicholson AG Yatabe Y Austin JHM Beasley MB . The 2015 World Health Organization classification of lung tumors: impact of genetic, clinical and radiologic advances since the 2004 classification. J Thorac Oncol. (2015) 10:1243–60. doi: 10.1097/JTO.0000000000000630 26291008

[12] Shih AR Mino-Kenudson M . Updates on spread through air spaces (STAS) in lung cancer. Histopathology. (2020) 77:173–80. doi: 10.1111/his.14062 31943337

[13] Funai K Sugimura H Morita T Shintani Y Matsuo K Okumura N . Lymphatic vessel invasion is a significant prognostic indicator in stage IA lung adenocarcinoma. Ann Surg Oncol. (2011) 18:2968–72. doi: 10.1245/s10434-011-1729-9 21512862

[14] Samejima J Yokose T Ito H Nakayama H Tsuboi M Yatabe Y . Prognostic significance of blood and lymphatic vessel invasion in pathological stage IA lung adenocarcinoma in the 8th edition of the TNM classification. Lung Cancer. (2019) 137:144–8. doi: 10.1016/j.lungcan.2019.09.022 31593845

[15] Kudo Y Saji H Shimada Y Matsubayashi J Nagao T Kakihana M . Proposal on incorporating blood vessel invasion into the T classification parts as a practical staging system for stage I non-small cell lung cancer. Lung Cancer. (2013) 81:187–93. doi: 10.1016/j.lungcan.2013.04.016 23664031

[16] Miyoshi K Moriyama S Kunitomo T Nawa S . Prognostic impact of intratumoral vessel invasion in completely resected pathologic stage I non-small cell lung cancer. J Thorac Cardiovasc Surg. (2009) 137:429–34. doi: 10.1016/j.jtcvs.2008.07.007 19185165

[17] Tsuchiya T Akamine S Muraoka M Kamohara R Tsuji H Urabe S . Stage IA non-small cell lung cancer: vessel invasion is a poor prognostic factor and a new target of adjuvant chemotherapy. Lung Cancer. (2007) 56:341–8. doi: 10.1016/j.lungcan.2007.01.019 17350137

[18] Ikeda T Kadota K Go T Misaki N Haba R Shimizu K . Segmentectomy provides comparable outcomes to lobectomy for stage IA non-small cell lung cancer with spread through air spaces. Semin Thorac Cardiovasc Surg. (2023) 35:156–63. doi: 10.1053/j.semtcvs.2022.02.001 35149218

[19] Chae M Jeon JH Chung JH Lee SY Hwang HJ Kim H . Prognostic significance of tumor spread through air spaces in patients with stage IA part-solid lung adenocarcinoma after sublobar resection. Lung Cancer. (2021) 152:21–6. doi: 10.1016/j.lungcan.2020.12.001 33338924

[20] Choi SH Jeong JY Lee SY Shin DM Lee S Lee H . Clinical implication of minimal presence of solid or micropapillary subtype in early-stage lung adenocarcinoma. Thorac Cancer. (2021) 12:235–44. doi: 10.1111/1759-7714.13754 33231358 PMC7812076

[21] Collins GS Reitsma JB Altman DG Moons KGM . Transparent reporting of a multivariable prediction model for individual prognosis or diagnosis (TRIPOD): the TRIPOD statement. BMJ. (2015) 350:g7594. doi: 10.1136/bmj.g7594 25569120

[22] Ito H Nakayama H Murakami S Yokose T Yamada K Masuda M . Does the histologic predominance of pathological stage IA lung adenocarcinoma influence the extent of resection? Gen Thorac Cardiovasc Surg. (2017) 65:512–8. doi: 10.1007/s11748-017-0790-0 28593420

[23] Peng B Li G Guo Y . Prognostic significance of micropapillary and solid patterns in stage IA lung adenocarcinoma. Am J Transl Res. (2021) 13:10562–9. PMC8507014 34650727

[24] Moreira AL Ocampo PSS Xia Y Zhong H Russell PA Minami Y . A grading system for invasive pulmonary adenocarcinoma: a proposal from the International Association for the Study of Lung Cancer pathology committee. J Thorac Oncol. (2020) 15:1599–610. doi: 10.1016/j.jtho.2020.06.001 32562873 PMC8362286

[25] Jeon HW Kim YD Sim SB Moon MH . Significant difference in recurrence according to the proportion of high grade patterns in stage IA lung adenocarcinoma. Thorac Cancer. (2021) 12:1952–8. doi: 10.1111/1759-7714.13984 34037324 PMC8258359

[26] Wang S Xu J Wang R Qian F Yang W Qiao R . Adjuvant chemotherapy may improve prognosis after resection of stage I lung cancer with lymphovascular invasion. J Thorac Cardiovasc Surg. (2018) 156:2006–2015.e2. doi: 10.1016/j.jtcvs.2018.06.034 30104070

[27] Ettinger DS Wood DE Aisner DL Akerley W Bauman JR Bharat A . Non-small cell lung cancer, version 3.2022, NCCN clinical practice guidelines in oncology. J Natl Compr Canc Netw. (2022) 20:497–530. doi: 10.6004/jnccn.2022.0025 35545176

[28] Pathak R Goldberg SB Canavan M Herrin J Hoag JR Marino K . Association of survival with adjuvant chemotherapy among patients with early-stage non-small cell lung cancer with vs without high-risk clinicopathologic features. JAMA Oncol. (2020) 6:1741–50. doi: 10.1001/jamaoncol.2020.4232 32940636 PMC7499246

[29] Su H Xie H Dai C Lin L Li W Chen Y . Procedure-specific prognostic impact of micropapillary subtype may guide resection strategy in small-sized lung adenocarcinomas: a multicenter study. Ther Adv Med Oncol. (2020) 12:1758835920937893. doi: 10.1177/1758835920937893 32670422 PMC7336827

