# Supplementary material for: An integrated multimodal model for early prediction of high-risk recurrence phenotype indicative of poor disease-free survival in stage IA NSCLC: a multicenter study
Source: Front Immunol. 2026 Aug 6;17:1915946. doi: 10.3389/fimmu.2026.1915946 (PMC13489873; doi:10.3389/fimmu.2026.1915946)
Supplement: Supplementary file 1 [file DataSheet1.docx]

**Supplementary Material**

This supplementary material accompanies the manuscript "An Integrated Multimodal Model for Early Prediction of High-Risk Recurrence Phenotype Indicative of Poor Disease-Free Survival in Stage IA NSCLC: A Multicenter Study." The information below details the model construction pipeline and is intended to be reviewed together with the main text.

# **Appendix. Model construction pipeline**

## **1. Clinical prediction model**

The clinical prediction model was developed based on preoperative clinical characteristics and CT morphological features, with data preprocessing, feature engineering, and model construction implemented as follows.

## **1.1 Feature inclusion**

A total of 16 features were included in the initial feature set, including:

• Demographic and clinical characteristics: gender (1 = male, 2 = female), age, ECOG PS score, number of preoperative tumors, maximum diameter of preoperative tumor (cm).

• CT morphological features: lobulation (1 = present, 0 = absent), spiculation (1 = present, 0 = absent), pleural indentation (1 = present, 0 = absent), vacuole sign (1 = present, 0 = absent), calcification (1 = present, 0 = absent), vascular convergence sign (1 = present, 0 = absent), nodule type (1 = ground-glass, 2 = solid, 3 = mixed), consolidation-to-tumor (C/T) ratio.

• Categorical encoding: the nodule type variable was converted into three dummy variables (ground-glass, solid, mixed) for model input.

## **1.2 Data preprocessing**

To ensure data quality and model robustness, the following preprocessing steps were performed:

• Numeric feature cleaning: custom extraction functions were applied to parse valid numeric values from raw unstructured data, including extracting the maximum value for tumor diameter, converting percentage values to decimals for C/T ratio, and extracting valid integers for tumor count.

• Binary feature processing: all binary morphological features were clipped to the range [0,1], with missing values filled with 0.

• Missing value imputation: median imputation was applied to all continuous numeric features to handle missing data.

• Feature standardization: all features were standardized via Z-score normalization (mean = 0, standard deviation = 1), with the scaler fitted only on the training set to avoid information leakage.

• Outcome encoding: the risk-group endpoint was encoded as a binary variable, where high-risk (original value = 1) was labeled as 1 and low-risk (original value = 2) was labeled as 0. Samples with missing outcome values were excluded from the analysis.

## **1.3 Model construction and training**

• Dataset split: the full dataset was split into a training set (70%) and a test set (30%) via stratified random sampling to maintain consistent class distribution between sets, with a fixed random seed of 42 for reproducibility.

• Classifier: an L2-regularized logistic regression model was used for risk prediction, with the following hyperparameters: solver = liblinear, regularization strength C = 1.0, maximum iteration = 1000, random state = 42.

• Feature selection: feature importance was ranked by the absolute value of the model coefficients, and features with non-zero coefficients were retained as the final clinical feature signature for downstream modeling.

## **2. CT radiomics model**

The CT radiomics model was developed based on quantitative features extracted from preoperative CT images, with feature extraction, preprocessing, and model construction implemented as follows.

## **2.1 Radiomics feature extraction**

Radiomics features were extracted from CT images and corresponding tumor regions of interest (ROI) using the pyradiomics (v3) toolbox.

• Extraction settings: binWidth was set to 25, the interpolator was set to sitkBSpline, and no resampling was performed (resampledPixelSpacing = None).

• Image type: original CT images were used for feature extraction.

• Feature classes: a total of 7 categories of radiomics features were extracted, including first-order statistics; shape features; Gray Level Co-occurrence Matrix (GLCM); Gray Level Run Length Matrix (GLRLM); Gray Level Size Zone Matrix (GLSZM); Gray Level Dependence Matrix (GLDM); and Neighboring Gray Tone Difference Matrix (NGTDM).

## **2.2 Feature preprocessing and model training**

• Dataset split: the dataset was split into a training set (70%) and a test set (30%) via stratified random sampling at the patient level, with a fixed random seed of 42. All feature preprocessing and model fitting were performed only on the training set to avoid information leakage.

• Feature standardization: Z-score normalization was applied to all radiomics features using StandardScaler, with the scaler fitted exclusively on the training set.

• Data augmentation: Gaussian noise (mean = 0, standard deviation = 0.1) was added to the standardized training set features to improve model generalization.

• Classifier: a support vector machine (SVM) with a radial basis function (RBF) kernel was used for radiomics-based risk prediction. Hyperparameters were set as follows: C = 1.0, gamma = 'scale', probability estimation enabled, class_weight = 'balanced' to address class imbalance, random state = 42, maximum iteration = 1000.

## **3. Pathology (WSI) deep learning model**

The pathology model was developed based on deep learning features extracted from whole-slide imaging (WSI) of hematoxylin–eosin (H&E)-stained pathological sections, with feature extraction and model construction implemented as follows.

## **3.1 WSI feature extraction**

• WSI preprocessing: foreground tissue regions were first segmented from raw WSIs to remove invalid background areas. The foreground regions were then tiled into non-overlapping patches under 20× magnification.

• Deep feature extraction: the UNI pathology foundation model was applied to extract high-dimensional feature vectors for each patch, generating patch-level deep representations of the pathological tissue.

## **3.2 Model construction and patient-level prediction**

• Dataset split: the dataset was split into a training set (70%) and a test set (30%) via stratified random sampling at the patient level, with a fixed random seed of 42. Patches from the same patient were exclusively assigned to either the training or test set to prevent data leakage.

• Feature standardization: Z-score normalization was applied to patch-level features, with the scaler fitted only on patches from the training set. GPU acceleration via the cuML library was supported to improve computational efficiency.

• Patch-level model training: an SVM model with an RBF kernel was trained on the standardized patch-level features. Hyperparameters were set as follows: C = 1.0, gamma = 'scale', probability estimation enabled, class_weight = 'balanced', random state = 42, maximum iteration = 1000.

• Patient-level inference: for each patient, the predicted high-risk probability of all patches belonging to the patient was averaged to obtain the patient-level risk probability. A classification threshold of 0.5 was applied to generate the final binary risk prediction for each patient.

## **4. Multimodal integrated machine learning model**

This model was designed to integrate complementary information from pathological WSI features, CT radiomics features, and clinical features via feature-level concatenation, to achieve comprehensive preoperative risk stratification. This approach uses multimodal feature-dimensional splicing to combine heterogeneous data sources, rather than methodologically defined multimodal fusion algorithms, which fully aligns with the actual implementation of the model. The model construction pipeline is detailed as follows.

## **4.1 Feature-level multimodal integration strategy**

Feature-level concatenation was adopted to combine complementary information from the three modalities. For each patient, multimodal integrated features were constructed at the patch level:

• WSI feature enhancement: the original patch-level WSI features were concatenated with log-transformed absolute value features (log1p(|feature|)) to enhance feature representation.

• Multimodal feature concatenation: the patient-level CT radiomics features and clinical features were tiled to match the number of patches of the corresponding patient, then concatenated with the enhanced WSI features to form the final patch-level multimodal integrated feature vector.

## **4.2 Informative patch sampling**

To reduce computational burden while preserving the most predictive information, an importance-based sampling strategy was applied:

• Proxy model training: a logistic regression model was trained as a proxy model on a random subset (max 200,000 samples) of the integrated training features, to evaluate the predictive value of each patch.

• Top-k sampling: for each patient, patches were ranked by the predicted risk probability from the proxy model, and the top 500 patches with the highest information content were retained for final integrated model training.

## **4.3 Integrated model construction and training**

• Feature standardization: Z-score normalization was applied to the sampled integrated features, with the scaler fitted only on the training set. GPU acceleration via the cuML library was supported.

• Main classifier: an SVM model with an RBF kernel was used as the main classifier, with hyperparameters set as: C = 10, gamma = 'scale', class_weight = 'balanced', probability output disabled, maximum iteration = 5000. The model output the decision value for each patch.

• Probability calibration: a logistic regression calibrator was trained on the decision values of the main SVM model, to convert decision values into calibrated risk probabilities.

• Patient-level inference: for each patient, the calibrated probabilities of all retained patches were averaged to obtain the final patient-level risk probability. A classification threshold of 0.5 was applied to generate binary risk predictions.

• Dataset split: the dataset was split into a training set (70%) and a test set (30%) via stratified random sampling at the patient level, with a fixed random seed of 42. All feature processing, sampling, and model fitting were performed exclusively on the training set to avoid information leakage.

## **5. Model evaluation metrics**

All models were evaluated on both the training set and independent test set, with the following core binary classification metrics calculated:

• Area under the receiver operating characteristic curve (AUC).

• Accuracy.

• Sensitivity (recall).

• Specificity.

• F1-score.

• Precision.

• Confusion matrix (true negative, false positive, false negative, true positive).

To assess the stability and statistical reliability of model performance, 2,000 iterations of bootstrap resampling were performed to calculate the 95% confidence interval (CI) for each metric, with a fixed random seed of 42 for reproducibility.
